# Role of 3D left ventricular end-systolic volume in risk stratification and outcome prediction in significant mitral regurgitation

**DOI:** 10.1093/ehjimp/qyag016

**Published:** 2026-01-28

**Authors:** Dana Cramariuc, Christian E Berg-Hansen, Lisa M D Grymyr, Rasmus Bach Sindre, Cecilie Linn Aas, Nina Ajmone Marsan, Judy Hung, Stig Urheim

**Affiliations:** Department of Heart Disease, Haukeland University Hospital, Jonas Lies vei 65, Bergen NO-5021, Norway; Department of Clinical Science, University of Bergen, Postbox 7804, Bergen NO-5020, Norway; Department of Heart Disease, Haukeland University Hospital, Jonas Lies vei 65, Bergen NO-5021, Norway; Department of Clinical Science, University of Bergen, Postbox 7804, Bergen NO-5020, Norway; Department of Heart Disease, Haukeland University Hospital, Jonas Lies vei 65, Bergen NO-5021, Norway; Department of Clinical Science, University of Bergen, Postbox 7804, Bergen NO-5020, Norway; Department of Clinical Science, University of Bergen, Postbox 7804, Bergen NO-5020, Norway; Department of Heart Disease, Haukeland University Hospital, Jonas Lies vei 65, Bergen NO-5021, Norway; Department of Cardiology, Leiden University Medical Center, Leiden, the Netherlands; Division of Cardiology, Cardiac Ultrasound Laboratory, Massachusetts General Hospital, Harvard Medical School, Boston, MA, USA; Department of Heart Disease, Haukeland University Hospital, Jonas Lies vei 65, Bergen NO-5021, Norway; Department of Clinical Science, University of Bergen, Postbox 7804, Bergen NO-5020, Norway

**Keywords:** mitral regurgitation, 3D echocardiography, left ventricular volumes, left ventricular dilatation, mitral valve

## Abstract

**Aims:**

In the follow-up of patients with mitral regurgitation (MR), assessment of left ventricular (LV) dilatation using standard echocardiography often yields inconsistent results. We investigated whether measuring 3D LV end-systolic volume (3DLVESV) improves risk stratification in moderate or greater MR.

**Methods and results:**

In the prospective 3D Echocardiography and Cardiovascular Prognosis in Mitral Regurgitation (3D-PRIME) study, 227 patients −142 with primary (PMR) and 85 secondary MR (SMR)- underwent 2D/3D echocardiography. 3DLVESV was increased if ≥41.5/35 mL/m², and LV end-systolic diameter (LVESD) enlarged if ≥39.8/34.8 mm in men/women. The primary outcome was a composite of MR progression towards intervention, death, or heart failure hospitalization (HFH). Death or HFH was a secondary outcome.

At baseline, 28% of PMR and 54% of SMR patients had increased 3DLVESV. After 21 (15–25) months, increased 3DLVESV was associated with 1.9-fold (1.2–3.2) higher adjusted risk of the primary outcome in PMR, and 4.1-fold (1.6–10.7) higher risk of death or HFH in SMR (*P* < 0.05). 3DLVESV and LVESD concordantly identified LV dilatation in 20% of PMR patients and were discordant in 27%. Both patients with increased 3DLVESV only, and those with increased both 3DLVESV and LVESD, had high risk of the primary outcome after adjusting for recommendations for intervention in PMR: HR 7.1 (2.9–16.9) and 4.9 (2.1–11.1), respectively (*P* < 0.001).

**Conclusion:**

Increased 3DLVESV is associated with a higher risk of adverse events in patients with significant MR. In PMR, evaluating LV dilatation using both 3DLVESV and LVESD may enhance risk stratification and aid in patient selection for close follow-up.

**ClinicalTrials.gov identifier:**

NCT04442828, 17 April 2020.

## Graphical Abstract

**Graphical Abstract:**
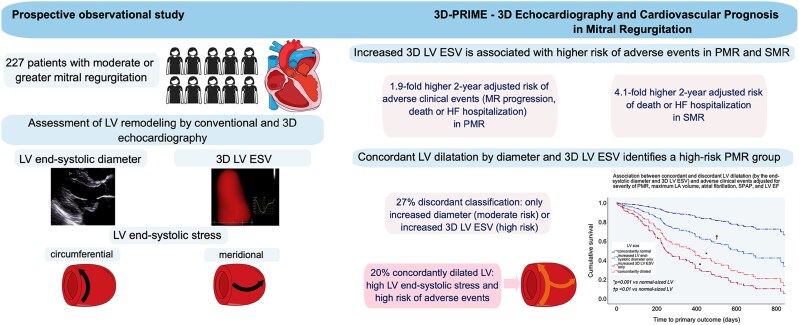
Increased 3D LV ESV and clinical outcomes in moderate or greater MR

## Introduction

Chronic mitral regurgitation (MR) causes gradual remodelling of the left ventricle (LV) with progressive dilatation, increased systolic wall stress and ultimately, if left untreated, LV decompensation.^1^ Once established, LV failure begets MR through distortion of the mitral valve apparatus.^2^ The severity of LV remodelling is an important component of the standard echocardiographic evaluation and influences the management of patients with both primary and secondary MR.^3^ As such, in patients with MR due to mitral leaflet pathology (primary MR), an increased LV end-systolic diameter ≥40 mm indicates the need of mitral valve intervention, even in the absence of symptoms. In patients with MR due to LV or left atrial (LA) dysfunction (secondary MR), the severity of LV dilatation must be carefully considered when evaluating the potential benefits or futility of treatment.^3^

The recommendation to assess LV dilatation using the inner end-systolic diameter stems from a large registry study of patients with flail mitral leaflets, which demonstrated an association between the M-mode-measured diameter and increased mortality risk.^4^ Subsequently, LV end-systolic diameter, along with six other variables, was incorporated into a multiparametric score that was shown to predict the risk of death in MR due to mitral valve prolapse.^5^ However, assessment of LV remodelling through linear echocardiographic indices, while simple and practical, often yields inconsistent results in clinical practice.^6^ This issue can be mitigated by measuring LV volumes, which provide a more accurate representation of LV shape and size compared to linear measurements.^7^ Three-dimensional (3D) echocardiography may further refine the assessment of LV size by avoiding geometrical simplifications and reducing measurement variability.^8^ However, the impact of 3D LV volume measurement on risk stratification in patients with MR has not been previously explored.

In the prospective 3D Echocardiography and Cardiovascular Prognosis in Mitral Regurgitation (3D-PRIME) study, we investigated whether systematic assessment of LV dilatation using 3D end-systolic volume (ESV) in patients with primary or secondary MR enhances risk stratification and improves the prediction of adverse events compared to standard echocardiographic measurement of LV end-systolic diameter. Additionally, we examined whether groups of patients classified concordantly or discordantly as having a dilated LV by 3D ESV and LV diameter differ in clinical presentation and risk of adverse events during MR progression.

## Methods

### Study population

The study cohort consisted of patients with significant MR enrolled prospectively in the 3D-PRIME study between 2020 and 2024. Inclusion criteria were age ≥18 years, presence of significant primary or secondary MR, and acquisition of 3D echocardiographic loops with good temporal and spatial resolution.^9^ Exclusion criteria were previous mitral valve intervention, other severe valvular heart disease, hypertrophic cardiomyopathy, congenital heart disease, pulmonary embolism, constrictive pericarditis or incomplete echocardiographic datasets. Patients with a possible mixed MR aetiology were not included in the primary MR group. The patients were followed up for 2 years if they did not reach an indication for valve intervention, or up to mitral valve intervention or death.

Clinical data consisting of symptom status, comorbidities (including earlier episodes of atrial fibrillation), and daily medication were collected through patient interview and chart reviews. The heart rhythm was verified using 12-lead electrocardiograms at each study visit. The study was conducted in accordance with the revised Declaration of Helsinki. All patients gave written informed consent, and the study was approved by the regional ethics committee (2020/106848).

### Echocardiographic measurements

All the echocardiographic examinations were performed using the same equipment (Vivid E95), and the acquisitions were analysed at the study echo core laboratory at Haukeland University Hospital using Echopac workstations (both GE Vingmed Ultrasound, Norway).^9^ In patients with atrial fibrillation during the examination, we recorded 3D loops during reasonably regular rhythm (variation in R-R-interval <10%) with no stitching artefacts.

#### Ventricular size and function

We measured the 3D LV end-diastolic volume and ESV in 4-to-6 beats full-volume 3D acquisitions with a temporal resolution of 40 ± 8 vol/s.^9^ To identify patients with 3D LV dilatation, we applied the 3D ESV/body surface area thresholds of 41.5 mL/m^2^ for men and 35 mL/m^2^ for women identified by the European Association of Cardiovascular Imaging (EACVI) Normal Reference Ranges for Echocardiography (NORRE) study in 440 healthy White European subjects.^10^ LV size was also assessed by measuring the end-systolic diameter in standard parasternal long-axis views and defined as increased if ≥39.8 mm in men and ≥34.8 mm in women in line with the current recommendations for chamber quantification.^7^ All analyses focused on measures of LV end-systolic size, as these are less affected by preload compared to LV end-diastolic size, are more closely associated with the early development of LV systolic dysfunction, and have previously shown superior predictive value in patients with MR.^4,11^

LV end-systolic stress was calculated in the circumferential direction using a cylindrical model and the following formula: *P* × (*a*^2^) × (1+ (*b*^2^/*r*^2^))/(*b*^2^-*a*^2^) where *P* is the systolic blood pressure (BP) from a cuff sphygmomanometer, *a* is the LV end-systolic endocardial radius, *b* is the epicardial radius, and *r* is the midwall radius. We calculated LV meridional end-systolic stress using the following invasively validated formula: 0.668 × *P* × *a*/(LV posterior wall thickness × (1 + LV posterior wall thickness/2a)).^12^

#### LA size and estimated systolic pulmonary artery pressure

The main measure of LA size was the maximum LA volume measured in apical four- and two-chamber LA-focused views.^9^ The maximum tricuspid regurgitation gradient was used in the estimation of systolic pulmonary artery pressure (SPAP).

#### MR grading

MR was graded based on a combination of qualitative, semiquantitative, and quantitative criteria as currently recommended.^13^ Quantification included the regurgitant volume and the regurgitant fraction, and severe MR was defined as a regurgitant fraction above 50%.^3,13^

### Outcomes

We examined the association between increased 3D LV ESV and the primary outcome of the 3D-PRIME study, a composite of all-cause mortality, heart failure (HF) hospitalization and MR progression towards severe with guideline indication for intervention (either mitral valve surgery or percutaneous mitral valve repair) during follow-up. Secondary, increased 3D LV ESV was analysed in relation to either MR progression towards intervention or combined death or HF hospitalization. All clinical events were ascertained from medical records. Referral to mitral intervention was based strictly on current European guidelines, and the referring clinicians were blinded to the results of the 3D echocardiographic analyses performed at the study echo core laboratory.^3^

### Statistical analyses

Statistical analyses were performed in IBM SPSS Statistics 28.0 (IBM Corp., Armonk, NY, USA) and R 4.4.0. All analyses were conducted separately in patients with primary and secondary MR. Comparisons between groups with increased vs. normal 3D LV ESV were performed by χ^2^ test or Fisher’s exact test for categorical variables, and independent-samples *t*-tests or Mann–Whitney *U* tests for continuous variables with vs. without normal distribution. Comparisons between the four groups with concordantly or discordantly dilated LV based on combined assessment of 3D LV ESV and end-systolic diameter were done by ANOVA or Kruskal–Wallis test with Bonferroni posthoc analysis. Findings are reported as percentages, mean ± standard deviation (SD), or median (25th–75th percentile).

The impact of increased 3D LV ESV on the primary and secondary outcomes was evaluated in Kaplan–Meier survival analyses with log-rank test for the overall analysis, as well as in Cox proportional hazards analyses. In the survival analyses with composite endpoints, we analysed the time to first occurring event. In the Cox analyses, factors that were both clinically relevant and associated with the outcome at *P* < 0.1 in univariable models were further tested in multivariable models run with an enter procedure. We assessed the change in the global χ^2^ value when each measure of LV dilatation (increased end-systolic LV diameter and 3D LV ESV) was added to an elementary Cox model including the current guidelines-recommended indications for intervention in primary MR. Absence of significant collinearity between increased 3D LV ESV and increased LV end-systolic diameter was tested before running the likelihood ratio tests. Additionally, we evaluated the change in the Harrell C concordance statistic index between the three Cox models. Results are reported as hazard ratios with 95% confidence intervals (CI). A two-tailed *P* < 0.05 was considered significant in all analyses.

## Results

### Baseline clinical and echocardiographic characteristics

The clinical features of the cohort are summarized in *Table 1*. The cause of MR was primary in 63% of cases, with secondary MR accounting for the remaining 37%. The group with secondary MR was, on average, 8 years older, included a higher proportion of women (48% vs. 32%), and had, as expected, a higher prevalence of cardiovascular risk factors and comorbidities (*Table 1*).

**Table 1:** Baseline clinical characteristics of patients with primary or secondary MR and increased vs. normal 3D LV ESV

	Primary MR (*n* = 142)		*P* value	Secondary MR (*n* = 85)		*P* value
	Increased 3D LV ESV			Increased 3D LV ESV		
	Yes (*n* = 40)	No (*n* = 102)		Yes (*n* = 46)	No (*n* = 39)	
Age, years	64 (52–75)	68 (57–78)	0.371	75 (71–79)	77 (74–82)	0.087
Women	23%	36%	0.115	30%	64%	**0**.**002**
BMI, kg/m^2^	24.8 ± 3.4	25.6 ± 3.6	0.216	25.8 ± 3.8	24.6 ± 4.8	0.206
Heart rate, bpm	66 (57–78)	69 (60–77)	0.557	71 (64–82)	70 (63–87)	0.754
Systolic BP, mmHg	136 (122–153)	136 (127–152)	0.465	132 (120–150)	141 (130–155)	0.054
Diastolic BP, mmHg	79 (72–89)	81 (75–87)	0.295	79 (72–84)	84 (75–91)	**0**.**026**
Atrial fibrillation	8%	11%	0.555	50%	69%	0.073
History of hypertension	48%	40%	0.428	65%	67%	0.888
Diabetes mellitus	2.5%	1%	0.489	20%	0%	**0**.**003**
Coronary artery disease	28%	12%	**0**.**022**	48%	31%	0.110
Chronic kidney disease	13%	5%	0.111	27%	26%	0.915
Current medication						
ACEi, ARB or ARNI	53%	41%	0.222	83%	69%	0.147
Beta-blockers	50%	44%	0.527	80%	85%	0.614
Aldosterone antagonists	15%	8%	0.198	30%	15%	0.103
SGLT2 inhibitor	5%	1%	0.134	22%	8%	0.073
Diuretics	35%	24%	0.165	59%	64%	0.610

ACEi, angiotensin-converting enzyme inhibitor; ARB, angiotensin receptor blocker; ARNI, angiotensin receptor-neprilysin inhibitor; BMI, body mass index; BP, blood pressure; MR, mitral regurgitation; MRA, mineralocorticoid receptor antagonist; SGLT2i, inhibitor of sodium-glucose transport protein 2.

Data are presented as median (25th–75th percentiles), mean ± standard deviation, or as percentages.

The *P* values for comparisons between patients with increased vs. normal 3D LV ESV are based on χ^2^ test or Fisher’s exact test for categorical variables, and independent-samples *t*-test or Mann–Whitney *U* test for continuous variables with vs. without a normal distribution.

An increased 3D LV ESV was present in 38% of patients: 28% of those with primary MR and 54% of those with secondary MR. Patients with primary MR and an increased 3D LV ESV more frequently had coronary artery disease, while those with secondary MR and enlarged 3D LV ESV had a greater prevalence of diabetes than patients with normal 3D LV ESV (*P* < 0.05, *Table 1*). Daily medication was comparable between the LV size groups across both MR subtypes. Furthermore, patients with an increased 3D LV ESV presented with larger MR regurgitant volumes and more severe left-heart remodelling, including larger atrial and ventricular volumes and lower LV EF, than patients with normal 3D LV ESV irrespective of MR aetiology (*P* < 0.05, *Table 2*).

**Table 2:** Baseline echocardiographic characteristics of patients with primary or secondary MR and increased vs. normal 3D LV ESV

	Primary MR (*n* = 142)		*P* value	Secondary MR (*n* = 85)		*P* value
	Increased 3D LV ESV			Increased 3D LV ESV		
	Yes (*n* = 40)	No (*n* = 102)		Yes (*n* = 46)	No (*n* = 39)	
3D LV end-diastolic volume, mL	218 (189–253)	150 (124–183)	**<0**.**001**	194 (173–232)	120 (92–150)	**<0**.**001**
3D LV end-diastolic volume/BSA, mL/m^2^	112 (102–125)	80 (67–91)	**<0**.**001**	102 (91–116)	69 (51–77)	**<0**.**001**
3D LV ESV, mL	92 (82–106)	54 (46–70)	**<0**.**001**	109 (89–140)	51 (38–63)	**<0**.**001**
3D LV ESV/BSA, mL/m^2^	47 (44–53)	30 (26–35)	Na	57 (48–72)	29 (20–33)	Na
LV end-systolic diameter, mm	41 (36–46)	35 (31–38)	**<0**.**001**	47 (42–52)	35 (31–39)	**<0**.**001**
LV end-systolic diameter/BSA, mm/m^2^	21 (19–23)	18 (16–20)	**<0**.**001**	25 (22–27)	19 (17–21)	**<0**.**001**
Maximum 2D LA volume, mL	115 (80–144)	83 (67–112)	**0**.**001**	123 (83–175)	96 (76–126)	**0**.**031**
Biplane EF, %	60 (54–63)	63 (58–67)	**0**.**005**	42 (36–49)	56 (53–62)	**<0**.**001**
LV GLS, %	17.8 (15.0–21.4)	18.8 (17.1–21.0)	0.315	10.3 (7.9–14.8)	14.6 (12.7—17.3)	**<0**.**001**
SPAP, mmHg	34 (25–42)	32 (10–40)	0.301	40 (30–44)	38 (32–46)	0.618
MR regurgitant volume (PISA), mL	72 (50–94)	45 (26–65)	**<0**.**001**	38 (28–46)	31 (21–40)	**0**.**003**
MR regurgitant fraction, %	61 (41–73)	49 (32–67)	**0**.**026**	51 (41–57)	44 (31–57)	0.161
Meridional LV end-systolic stress, dyne/cm^2^	120 (96–152)	102 (84–127)	**0**.**002**	169 (114–143)	96 (79–134)	**<0**.**001**
Circumferential LV end-systolic stress, dyne/cm^2^	148 (123–186)	133 (117–157)	**0**.**019**	197 (143–234)	126 (108–168)	**<0**.**001**

Data are presented as median (25th–75th percentiles).

The *P* values for comparisons between patients with increased vs. normal 3D LV ESV are based on Mann–Whitney *U* tests.

### 3D LV dilatation in relation to clinical outcomes

During a median follow-up of 21 (15–25) months, there were 53 mitral valve surgeries, 46 percutaneous mitral valve repairs, 21 hospitalizations for HF, and 20 deaths in the entire cohort. Among the 99 patients referred to intervention, the majority (*n* = 78, i.e. 79%) had progression of MR to severe with symptom onset, while 21 patients (21%) experienced increasing LV diameter above 40 mm or reduction in EF during follow-up. Among patients with an increased 3D LV ESV at baseline, 65% experienced adverse events, and 28% died or were hospitalized for HF. In contrast, the corresponding rates among patients with normal 3D LV ESV were 45% and 11%, respectively (both *P* < 0.01).

**Figure 1:**
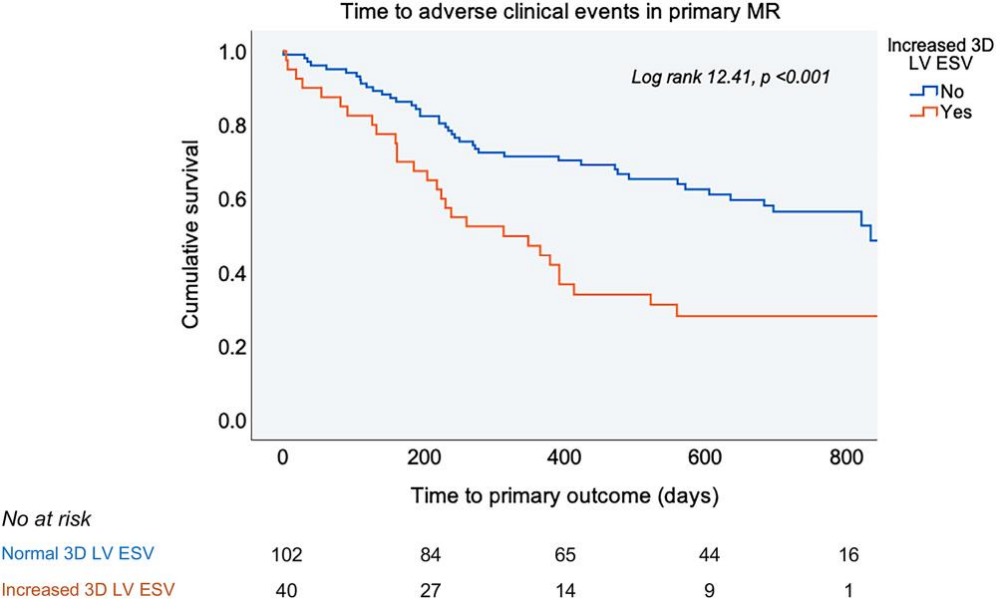
Kaplan-Meier curves for time to first adverse clinical event (MR progression towards intervention, death or HF hospitalization) in patients with primary MR and increased or normal 3D LV ESV. The value of the log rank test is presented for the overall analysis with the respective *P* value.

**Figure 2:**
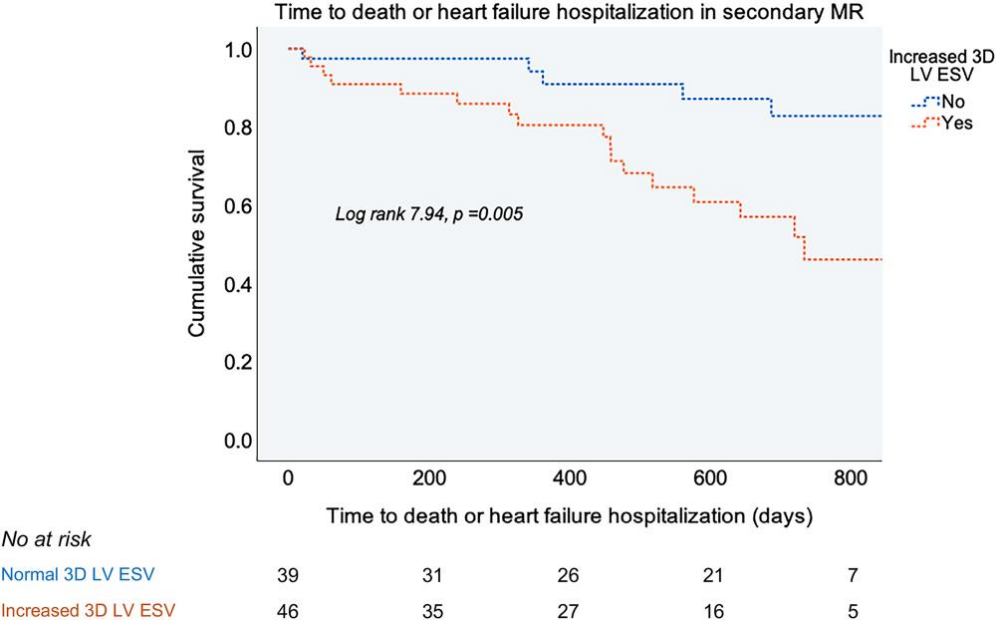
Kaplan-Meier curves for time to death and HF hospitalization in patients with secondary MR and increased or normal 3D LV ESV. The value of the log rank test is presented for the overall analysis with the respective *P* value.

In univariable Cox analyses, larger 3D LV ESV predicted the primary composite outcome in both primary and secondary MR with a similar hazard ratio (HR) of 1.01 [95% CI (1.00–1.02), *P* < 0.05]. When thresholds for 3D dilatation were applied, an increased 3D LV ESV was associated with a 2.3-fold higher risk of the primary endpoint and a 2.6-fold higher risk of MR progression towards intervention among patients with primary MR (*Table 3*, *Figure 1*). In patients with secondary MR, an increased 3D LV ESV did not predict the primary outcome but was related to 3.5-fold higher risk of death or HF hospitalization (*Table 3*, *Figure 2*). These associations did not change after adjustment for age and MR severity in multivariable Cox analyses (*Table 3*).

**Table 3:** Clinical outcomes at 2 years follow-up in patients with increased vs. normal 3D LV ESV

Primary MR	Increased 3D LV ESV (*n* = 40)	Normal 3D LV ESV (*n* = 102)	HR [95% CI]	*P* value	Adjusted HR [95% CI] *	*P* value
Adverse events (MR progression towards intervention, death, or HF hospitalization)	28 (70%)	46 (45%)	2.32 [1.43–3.75]	**<0.001**	1.92 [1.17–3.16]	**0**.**010**
MR progression towards intervention	28 (70%)	40 (39%)	2.62 [1.60–4.29]	**<0**.**001**	2.08 [1.25–3.47]	**0**.**005**
Death or HF hospitalization	5 (13%)	9 (9%)	2.26 [0.75–6.83]	0.148	2.54 [0.79–8.17]	0.118

Secondary MR	Increased 3D LV ESV (*n* = 46)	Normal 3D LV ESV (*n* = 39)	HR [95% CI]	*P* value	Adjusted HR [95% CI]^a^	*P* value
Adverse events (MR progression towards intervention, death, or HF hospitalization)	28 (61%)	17 (44%)	1.73 [0.94–3.17]	0.078	1.70 [0.88–3.31]	0.118
MR progression towards intervention	17 (37%)	14 (36%)	1.15 [0.56–2.33]	0.709	0.95 [0.44–2.06]	0.900
Death or HF hospitalization	19 (41%)	6 (15%)	3.48 [1.38–8.74]	**0**.**008**	4.10 [1.58–10.65]	**0**.**004**

Data are reported as *n* (%) and HR [95% CI].

^a^Adjusted for age and MR severity.

### Combined assessment of LV end-systolic size by diameter and 3D volume and relation to clinical outcomes

At study enrolment, 51% of the entire cohort- 38% of patients with primary MR and 72% of those with secondary MR- presented with an increased LV end-systolic diameter. Only 28 patients with primary MR (20%) had concordantly classified LV as dilated by both 3D LV ESV and end-systolic diameter, while 38 patients (27%) were discordantly classified (*P* < 0.001). Patients with primary MR and concordantly dilated LV presented with comparable MR severity but a higher burden of coronary artery disease (32%) and higher use of antihypertensive and antidiabetic medication (75% and 11%, respectively) than those with discordantly classified or normal-sized LV (all *P* < 0.05). They also exhibited the highest circumferential [148 (118–187) dyne/cm^2^] and meridional LV end-systolic stress [123 (95–153) dyne/cm^2^] within the primary MR group (both *P* < 0.05), while discordant patients showed intermediate stress values (see Supplementary data online, *Figure S1*).

**Figure 3:**
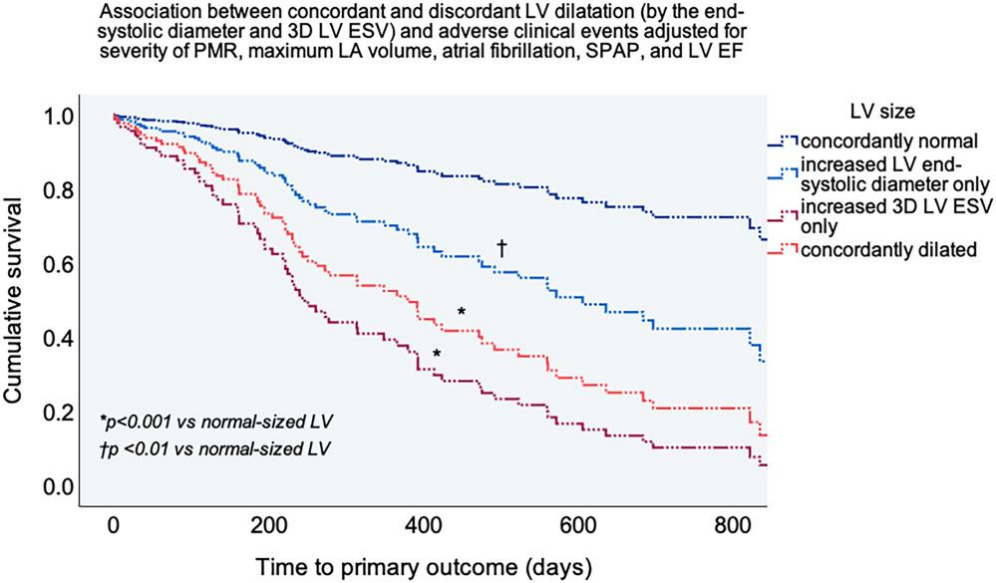
The adjusted association between concordantly or discordantly classified LV size (based on 3D LV ESV and LV end-systolic diameter) and adverse clinical events in primary MR. The *P* values show the significance of the difference between different categories of dilated LV vs. concordantly normal-sized LV after adjustment for MR severity, maximum LA volume, atrial fibrillation, SPAP and LV EF in multivariable Cox analysis.

**Figure 4:**
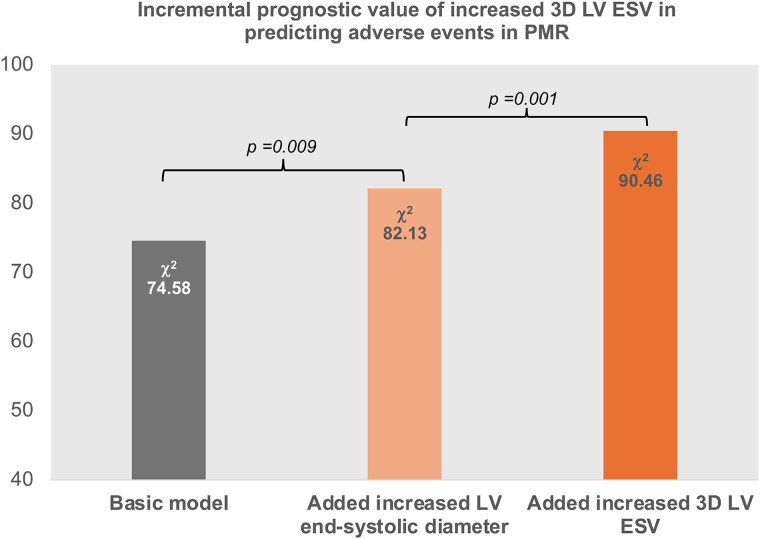
Likelihood ratio tests for the incremental value of increased 3D LV ESV in predicting adverse clinical events in primary MR over a basic clinical model (including MR severity, maximum LA volume, LV EF, atrial fibrillation, and SPAP) and over the basic model plus increased LV end-systolic diameter.

In multivariable Cox analysis including current guideline-based recommendations for intervention in primary MR, both isolated 3D LV dilatation (increased 3D LV ESV only) and concordantly dilated LV (increased both 3D ESV and end-systolic diameter) were strongly associated with a higher adjusted risk of clinical events (*Table 4*, *Figure 3*). Patients with primary MR and increased LV diameter only had a higher adjusted risk of events compared to those with concordantly normal LV size (*P* < 0.05) but fared better than the groups with increased 3D LV ESV (*Figure 3*). Further adjustments for age, sex, symptoms, coronary artery disease, or daily medication did not alter the results. Furthermore, adding first the increased LV end-systolic diameter and subsequently increased 3D LV ESV to a basic model incorporating current recommendations for intervention in primary MR gradually increased the model’s χ^2^ value (*Figure 4*). This was corroborated by the Harrell C concordance statistic, that showed a slight rise in the C-index when adding increased LV end-systolic diameter to the basic clinical model (from 0.773 to 0.800, *P* = 0.07), but a significant improvement upon incorporating the increased 3D LV ESV (C-index 0.817, *P* = 0.004), confirming the incremental prognostic value of this measure of LV dilatation.

**Table 4:** Adjusted association between concordantly and discordantly dilated LV (based on increased 3D LV ESV and LV end-systolic diameter) and adverse clinical events in patients with primary MR

Variable	Wald	HR [95% CI]	*P*
Concordantly normal LV size	23.04		<0.0001
Increased LV end-systolic diameter only	8.36	2.67 [1.37–5.20]	0.004
Increased 3D LV ESV only	19.11	7.05 [2.94–16.92]	<0.001
Concordantly dilated LV	14.02	4.87 [2.13–11.14]	<0.001
Severe MR	19.25	5.00 [2.44–10.27]	<0.001
Maximum LA volume, mL	8.65	1.01 [1.00–1.02]	0.003
Systolic pulmonary artery pressure, mmHg	0.04	1.00 [0.99–1.02]	0.835
LV EF, %	15.44	1.10 [1.05–1.15]	<0.001
Atrial fibrillation	0.27	1.21 [0.59–2.50]	0.603

Data are reported as Wald and HR [95% CI].

In patients with secondary MR, an increased LV end-systolic diameter was related to 3.5 (95% CI [1.1–11.7], *P* < 0.05) higher risk of death or HF hospitalization. The rate of discordance between increased LV end-systolic diameter and increased 3D LV ESV was lower than in primary MR, at 22%, while concordance was higher, with 52% of patients having dilated LV by both measures. Patients with concordantly dilated LV had a 5.6 [95% CI (1.6–19.6), *P* < 0.01] higher hazard of death or HF hospitalization compared to patients with normal LV size, after adjusting for age and MR severity. The discordantly classified patients did not demonstrate a higher adjusted risk for this outcome.

## Discussion

In this prospective investigation, we explored the benefits of measuring 3D end-systolic volume alongside conventional end-systolic LV diameter for detecting LV dilatation, stratifying risk in patients with moderate or greater MR, and predicting adverse clinical outcomes over a 2-year follow-up. Our findings can be summarized as follows: (i) An increased 3D LV ESV was observed in more than one in four patients with significant primary MR and one in two patients with significant secondary MR. This increase in 3D LV ESV was associated with larger MR volumes, a greater burden of cardiovascular risk factors and disease, and more severe remodelling of the left heart chambers. (ii) In primary MR, an increased 3D LV ESV was linked to a 2.3-fold higher risk of adverse events during the 2-year follow-up. (iii) In secondary MR, an increased 3D LV ESV correlated with a 3.5-fold higher risk of death or HF hospitalization. (iv) The combined assessment of 3D LV ESV and LV end-systolic diameter enabled more nuanced risk prognostication in primary MR. Here, an increase in LV size in the longitudinal direction (assessed via 3D ESV) translated into a similar impairment in survival as observed with combined radial-longitudinal dilatation (i.e. concordant LV dilatation). In contrast, an isolated increase in diameter (radial LV dilatation) correlated with a more modest increase in the risk of MR progression, death, or HF hospitalization. Our data support the use of a combined assessment of LV end-systolic diameter and 3D LV ESV in moderate or greater MR, particularly in cases of primary MR.

Assessment of LV size using 3D echocardiography offers several advantages over traditional echocardiographic measurements, including the avoidance of LV foreshortening and geometric assumptions, improved reproducibility and greater agreement with cardiac magnetic resonance imaging.^14^ Moreover, it is significantly more accessible and sustainable than other 3D imaging modalities.^15^ Despite these benefits, assessment of LV size in patients with MR is still recommended performed by linear measurements by current guidelines.^3,16^ This recommendation is grounded in a seminal study from the MIDA registry which collected echocardiographic M-mode data from 1980 to 2004 and demonstrated that an increased LV internal diameter above 40 mm predicted poorer survival in patients with flail mitral leaflets.^4^ However, in clinical practice, relying solely on cavity diameter for assessing LV remodelling can lead to significant inconsistencies between evaluators and across examinations.^17^

The role of 3D echocardiographic LV volumes in risk stratification for patients with MR was unclear prior to our investigation. Over the past decade, normal values for 3D LV volumes have been proposed by various studies, including large multicentre investigations.^10,18–21^ We opted for a set of sex-specific reference values defined in a population ethnically similar to ours, considering the known effect of race on LV size and shape.^21,22^ These thresholds are higher than those reported in earlier studies of normal 3D LV size, likely reflecting advancements in 3D image resolution that allow for better differentiation between compact LV wall myocardium and endocardial trabeculae, thus reducing underestimation of true LV cavity size.^18,19,23^ In both MR aetiologies, the NORRE thresholds for 3D LV dilatation facilitated the identification of a subgroup of patients with increased 3D LV ESV and a high-risk baseline profile characterized by greater cardiovascular risk or established cardiovascular disease, enlarged LA, higher LV end-systolic stress, and lower LV systolic function. Survival analyses confirmed that these patients had a higher 2-year rate of adverse events, even after adjusting for baseline age and MR severity. Interestingly, in primary MR, an increased 3D LV ESV was primarily associated with a greater risk of MR progression, reflecting the lower proportion of patients with primary MR who experience death or HF hospitalization while under close surveillance at a Heart Valve Clinic.^3^ In secondary MR, the degree of MR, quantified by the regurgitant fraction (the preferred method of MR quantification, especially in secondary aetiology where low-flow status is often observed), was comparable between patients with normal or increased 3D LV ESV; however, an increased 3D LV ESV was associated with a notably higher risk of death or HF hospitalization. This finding underscores that ventricular remodelling in patients with secondary MR is complex and multifactorial, reflecting not only the severity of MR but also the underlying myocardial disease that contributed to the development of MR.^24^

The prevalence of LV dilatation was different when defined by LV end-systolic diameter than by 3D LV ESV in both primary and secondary MR, with the groups of dilated LVs overlapping only partially. Through the combined assessment of LV end-systolic diameter and 3D LV ESV, we delineated subgroups of patient with more spherically remodelled or radially dilated LVs (increased diameter but normal 3D ESV), tear-formed or longitudinally dilated ventricles (normal diameter, increased 3D ESV), and bidirectionally dilated LVs (concordantly increased diameter and 3D ESV).^25^ A recent analysis of 3D echocardiograms from the World Alliance Societies of Echocardiography study revealed significant heterogeneity in LV shape among healthy individuals.^22^ As such, the three predominant LV shapes identified were the apparent size model (characterized by concordant variations in the longitudinal and radial directions), the aspect ratio model (discordant variations in these same directions), and the LV apical tilt.^22^ This supports the notion that the LV may remodel pathologically in overload conditions following similar distinct patterns, as demonstrated in our study of patients with MR. In our population, 27% of patients had discordant LV dilatation, accompanied by a milder increase in LV end-systolic wall stress. Interestingly, our data indicate that increased 3D LV ESV only (i.e. a longitudinally dilated LV) is associated with a notably worse prognosis in primary MR, presenting a 7-fold higher adjusted risk of adverse outcomes during a 2-year follow-up compared to LVs of normal size. This finding may indicate unfavourable regional myocardial remodelling in response to increased wall stress in tear-formed ventricles, warranting further exploration in mechanistic studies.^26^ It may also reflect the greater sensitivity of 3D echocardiographic volumes in detecting LV remodelling compared to linear dimensions. On the other hand, simultaneous LV dilatation in both the radial and longitudinal directions (concordant dilatation by both diameter and 3D ESV) was accompanied by a substantial and global increase in end-systolic wall stress, likely signalling a transition from a compensated to a decompensated phase in chronic MR.^27^

Patients with secondary MR had, as expected, more advanced stages of myocardial disease and a high prevalence of LV dilatation. This may explain the high concordance in identifying LV dilatation between 3D LV ESV and LV end-systolic diameter, as most ventricles were dilated in both directions. Future prospective studies in larger populations with diverse phenotypes of secondary MR should further explore the role of 3D LV dilatation as a risk-stratifying and treatment-guiding tool.

### Clinical perspective

In patients with moderate or greater MR, incorporating 3D LV ESV into the standard echocardiographic protocol can help identify a high-risk group characterized by LV dilatation in both the radial and longitudinal directions. For patients with primary MR who present combined radial and longitudinal LV dilatation, stringent management of cardiovascular risk factors, along with a thorough evaluation of the benefits vs. risks of valve intervention by the Heart Valve Team, should not be delayed. Patients with primary MR and discordant phenotypes, particularly those with isolated longitudinal LV dilatation, may require more intensive follow-up until a guideline-based indication for valve intervention is established. Future randomized clinical trials should determine whether the identification of LV dilatation through 3D LV ESV should prompt mitral valve intervention in asymptomatic primary MR.

### Study limitations

The 3D-PRIME study was conducted at a single Heart Valve Centre by investigators experienced in 3D echocardiography and had a shorter follow-up duration of two years, or until mitral intervention or death, as predefined in the study protocol. As a result, its findings should be evaluated in larger cohorts with longer follow-up periods. However, the prospective design facilitated the use of a comprehensive 3D echocardiographic protocol and produced uniformly high-quality 3D acquisitions and analyses. The different patterns of LV remodelling identified by the combined assessment of LV end-systolic diameter and 3D LV ESV might partly reflect intrinsic geometric variability rather than transitions in disease severity. These findings are not yet mechanistically validated and require further confirmation using longitudinal remodelling analyses or independent imaging modalities such as cardiac magnetic resonance imaging. We included the entire 3D-PRIME cohort, encompassing both primary and secondary MR, but conducted analyses separately in these two groups as these pathologies are aetiologically distinct. Both primary and secondary MR are associated with chronic LV overload, and the assessment of LV size is important for managing both conditions. However, the secondary MR group was small, and the predictive value of 3D LV dilatation in patients with ventricular vs. atrial secondary MR needs further investigation in larger populations with extended follow-up times to ensure higher event rates. In 21 patients, referral to mitral valve intervention was based on progression of MR to severe accompanied by significant LV remodelling with increasing diameter above 40 mm or fall in LV EF during follow-up. In the present analyses, we defined LV dilatation based on baseline values of LV diameter, using thresholds of normality rather than intervention criteria. However, we cannot rule out some degree of residual confounding in these cases, as the LV end-systolic diameter was accessible to the clinician and influenced the decision to intervene.

## Conclusion

In patients with moderate or greater MR, an increased 3D LV ESV is associated with a higher risk of adverse events in both primary and secondary MR. Evaluating LV dilatation through both LV end-systolic diameter and 3D LV ESV, rather than relying solely on a single measure, allows for better identification of patients with primary MR at increased risk of MR progression, death, or HF hospitalization. Our findings lay the groundwork for improved patient selection and better-targeted interventions in significant MR.

## Supplementary Material

qyag016_Supplementary_Data

## Data Availability

The data that support the findings of this study are available from the corresponding author upon reasonable request.

## Lead author biography

Dana Cramariuc is a cardiologist and associate professor in the Department of Heart Disease, Haukeland University Hospital and the Department of Clinical Science, University of Bergen, Norway. Her current research focuses on 3D echocardiography, valvular heart diseases, and cardiac remodelling under chronic overload conditions. Dana serves as the board leader of the West-Norwegian Cardiology Association and heads the ValveSmart group at Haukeland University Hospital.

## References

[1] Gaasch WH, Meyer TE. Left ventricular response to mitral regurgitation: implications for management. Circulation 2008;118:2298–303. 19029478 10.1161/CIRCULATIONAHA.107.755942

[2] Lancellotti P, Garbi M. Progression of secondary mitral regurgitation: from heart failure to valvular heart failure. Eur Heart J Cardiovasc Imaging 2018;19:613–4. 29534168 10.1093/ehjci/jey040

[3] Praz F, Borger MA, Lanz J, Marin-Cuartas M, Abreu A, Adamo M et al 2025 ESC/EACTS Guidelines for the management of valvular heart disease. Eur J Cardiothorac Surg 2025;67:1–109.

[4] Tribouilloy C, Grigioni F, Avierinos JF, Barbieri A, Rusinaru D, Szymanski C et al Survival implication of left ventricular end-systolic diameter in mitral regurgitation due to flail leaflets a long-term follow-up multicenter study. J Am Coll Cardiol 2009;54:1961–8. 19909877 10.1016/j.jacc.2009.06.047

[5] Grigioni F, Clavel MA, Vanoverschelde JL, Tribouilloy C, Pizarro R, Huebner M et al The MIDA Mortality Risk Score: development and external validation of a prognostic model for early and late death in degenerative mitral regurgitation. Eur Heart J 2018;39:1281–91. 29020352 10.1093/eurheartj/ehx465

[6] Hagendorff A, Knebel F, Helfen A, Stobe S, Haghi D, Ruf T et al Echocardiographic assessment of mitral regurgitation: discussion of practical and methodologic aspects of severity quantification to improve diagnostic conclusiveness. Clin Res Cardiol 2021;110:1704–33. 33839933 10.1007/s00392-021-01841-y PMC8563569

[7] Lang RM, Badano LP, Mor-Avi V, Afilalo J, Armstrong A, Ernande L et al Recommendations for cardiac chamber quantification by echocardiography in adults: an update from the American Society of Echocardiography and the European Association of Cardiovascular Imaging. J Am Soc Echocardiogr 2015;28:1–39 e14. 25559473 10.1016/j.echo.2014.10.003

[8] Thavendiranathan P, Grant AD, Negishi T, Plana JC, Popovic ZB, Marwick TH. Reproducibility of echocardiographic techniques for sequential assessment of left ventricular ejection fraction and volumes: application to patients undergoing cancer chemotherapy. J Am Coll Cardiol 2013;61:77–84. 23199515 10.1016/j.jacc.2012.09.035

[9] Berg-Hansen CE, Sindre RB, Grymyr LMD, Rogge B, Valeur AE, Urheim S et al Sex differences in left atrial volumes, mechanics, and stiffness in primary mitral regurgitation-a combined 2D and 3D echocardiographic study. Eur Heart J Cardiovasc Imaging 2024;25:1118–26. 38469654 10.1093/ehjci/jeae072 PMC11288747

[10] Bernard A, Addetia K, Dulgheru R, Caballero L, Sugimoto T, Akhaladze N et al 3D echocardiographic reference ranges for normal left ventricular volumes and strain: results from the EACVI NORRE study. Eur Heart J Cardiovasc Imaging 2017;18:475–83. 28329230 10.1093/ehjci/jew284

[11] Topilsky Y, Essayagh B, Benfari G, Le Tourneau T, Antoine C, Grigioni F et al Disproportionate left ventricular enlargement in mitral valve prolapse: prevalence, predictors, and association with outcomes. J Am Heart Assoc 2025;14:e040868. 40673524 10.1161/JAHA.124.040868 PMC12449906

[12] de Simone G, Devereux RB, Roman MJ, Ganau A, Saba PS, Alderman MH et al Assessment of left ventricular function by the midwall fractional shortening/end-systolic stress relation in human hypertension. J Am Coll Cardiol 1994;23:1444–51. 8176105 10.1016/0735-1097(94)90390-5

[13] Zoghbi WA, Adams D, Bonow RO, Enriquez-Sarano M, Foster E, Grayburn PA et al Recommendations for noninvasive evaluation of native valvular regurgitation: a report from the American society of echocardiography developed in collaboration with the society for cardiovascular magnetic resonance. J Am Soc Echocardiogr 2017;30:303–71. 28314623 10.1016/j.echo.2017.01.007

[14] Lang RM, Addetia K, Narang A, Mor-Avi V. 3-Dimensional echocardiography: latest developments and future directions. JACC Cardiovasc Imaging 2018;11:1854–78. 30522687 10.1016/j.jcmg.2018.06.024

[15] Gunasekaran S, Szava-Kovats A, Battey T, Gross J, Picano E, Raman SV et al Cardiovascular imaging, climate change, and environmental sustainability. Radiol Cardiothorac Imaging 2024;6:e240135. 38900024 10.1148/ryct.240135 PMC11211952

[16] Otto CM, Nishimura RA, Bonow RO, Carabello BA, Erwin JP 3rd, Gentile F et al 2020 ACC/AHA guideline for the management of patients with valvular heart disease: a report of the American College of Cardiology/American Heart Association joint committee on clinical practice guidelines. Circulation 2021;143:e72–227. 33332150 10.1161/CIR.0000000000000923

[17] Delling FN, Noseworthy PA, Adams DH, Basso C, Borger M, Bouatia-Naji N et al Research opportunities in the treatment of mitral valve prolapse: JACC expert panel. J Am Coll Cardiol 2022;80:2331–47. 36480975 10.1016/j.jacc.2022.09.044 PMC9981237

[18] Aune E, Baekkevar M, Rodevand O, Otterstad JE. Reference values for left ventricular volumes with real-time 3-dimensional echocardiography. Scand Cardiovasc J 2010;44:24–30. 19626561 10.3109/14017430903114446

[19] Muraru D, Badano LP, Peluso D, Dal Bianco L, Casablanca S, Kocabay G et al Comprehensive analysis of left ventricular geometry and function by three-dimensional echocardiography in healthy adults. J Am Soc Echocardiogr 2013;26:618–28. 23611056 10.1016/j.echo.2013.03.014

[20] Chahal NS, Lim TK, Jain P, Chambers JC, Kooner JS, Senior R. Population-based reference values for 3D echocardiographic LV volumes and ejection fraction. JACC Cardiovasc Imaging 2012;5:1191–7. 23236967 10.1016/j.jcmg.2012.07.014

[21] Addetia K, Miyoshi T, Amuthan V, Citro R, Daimon M, Gutierrez Fajardo P et al Normal values of left ventricular size and function on three-dimensional echocardiography: results of the world alliance societies of echocardiography study. J Am Soc Echocardiogr 2022;35:449–59. 34920112 10.1016/j.echo.2021.12.004

[22] Zhao D, Addetia K, Slivnick JA, Asch FM, Quill GM, Young AA et al Normal variations in left ventricular shape from 3D echocardiography: insights from the WASE study. Eur Heart J 2025;46:1.39749540

[23] Zhao D, Quill GM, Gilbert K, Wang VY, Houle HC, Legget ME et al Systematic comparison of left ventricular geometry between 3D-echocardiography and cardiac magnetic resonance imaging. Front Cardiovasc Med 2021;8:728205. 34616783 10.3389/fcvm.2021.728205 PMC8488135

[24] Huang AL, Dal-Bianco JP, Levine RA, Hung JW. Secondary mitral regurgitation: cardiac remodeling, diagnosis, and management. Struct Heart 2023;7:100129. 37273859 10.1016/j.shj.2022.100129 PMC10236886

[25] Broch K, de Marchi SF, Massey R, Hisdal J, Aakhus S, Gullestad L et al Left ventricular contraction pattern in chronic aortic regurgitation and preserved ejection fraction: simultaneous stress-strain analysis by three-dimensional echocardiography. J Am Soc Echocardiogr 2017;30:422–30 e2. 28065583 10.1016/j.echo.2016.11.012

[26] de Marvao A, Dawes TJ, Shi W, Durighel G, Rueckert D, Cook SA et al Precursors of hypertensive heart phenotype develop in healthy adults: a high-resolution 3D MRI study. JACC Cardiovasc Imaging 2015;8:1260–9. 26476505 10.1016/j.jcmg.2015.08.007 PMC4639392

[27] Corin WJ, Monrad ES, Murakami T, Nonogi H, Hess OM, Krayenbuehl HP. The relationship of afterload to ejection performance in chronic mitral regurgitation. Circulation 1987;76:59–67. 3594776 10.1161/01.cir.76.1.59

