# Supplementary material for: Role of 3D left ventricular end-systolic volume in risk stratification and outcome prediction in significant mitral regurgitation
Source: Eur Heart J Imaging Methods Pract. 2026 Jan 28;4(1):qyag016. doi: 10.1093/ehjimp/qyag016 (PMC12888048; doi:10.1093/ehjimp/qyag016)
Supplement: qyag016_Supplementary_Data [file qyag016_supplementary_data.zip › Supplementary Figure.docx]

**Supplementary Figure.** LV end-systolic stress measured in circumferential and meridional direction in patients with primary MR and concordantly normal-sized LV, increased LV end-systolic diameter only, increased 3D LV ESV only, or concordantly dilated LV.

The p values in the blue/ yellow boxes show the significance of the difference between different categories of LV size. #p <0.01 vs. concordantly normal-sized LV by Bonferroni´s posthoc test.

ESV, end-systolic volume; LV, left ventricular; MR, mitral regurgitation
