# Supplementary figures and images for: Role of 3D left ventricular end-systolic volume in risk stratification and outcome prediction in significant mitral regurgitation
Source: Eur Heart J Imaging Methods Pract. 2026 Jan 28;4(1):qyag016. doi: 10.1093/ehjimp/qyag016 (PMC12888048; doi:10.1093/ehjimp/qyag016)

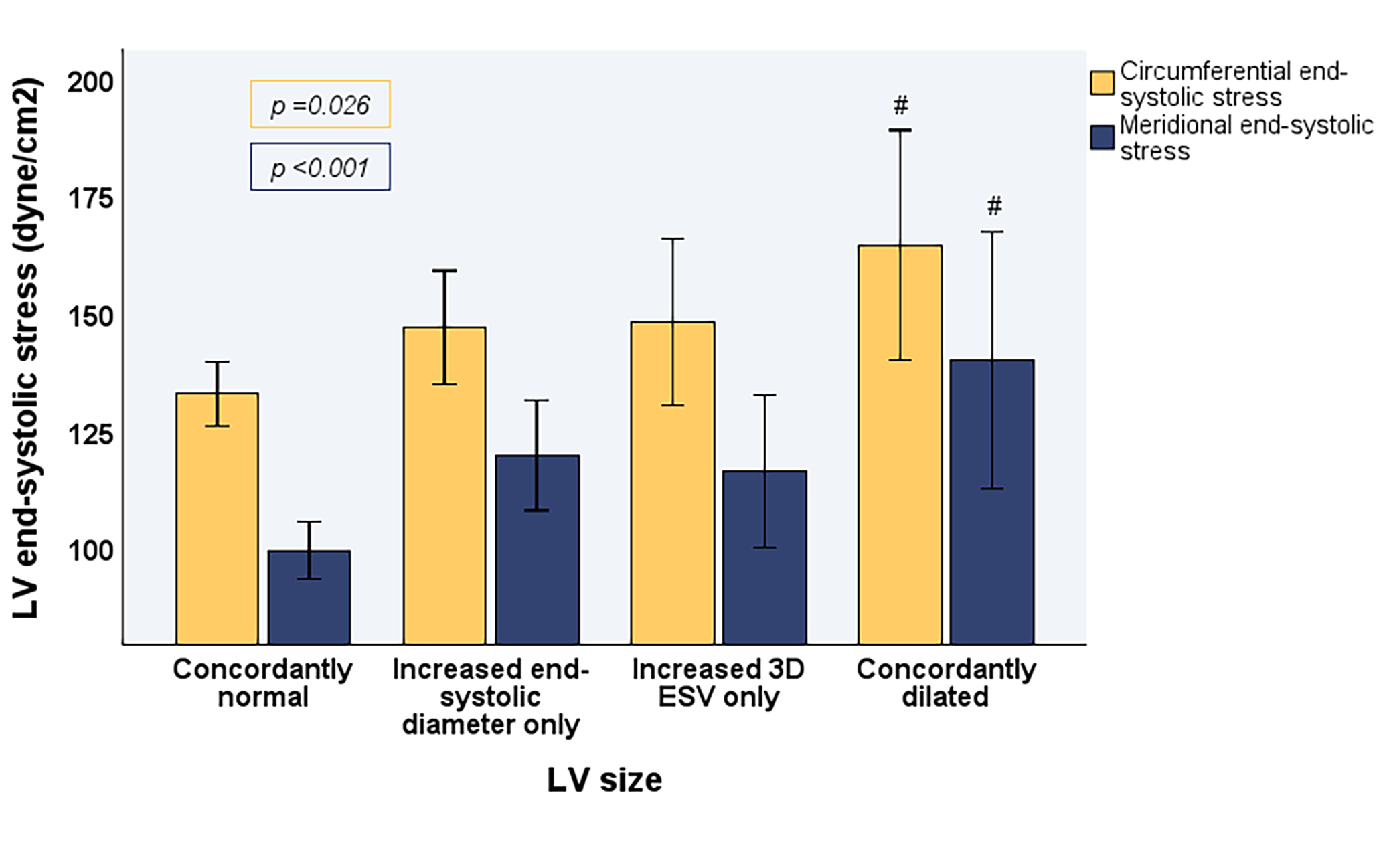

Supplement: qyag016_Supplementary_Data [file qyag016_supplementary_data.zip › Suppl figure rev Final.tif]
